# Introducing family medicine in Tanzania: strengthening primary health care through the 5 Cs—advocacy through a deliberative dialogue symposium-a case study of multinational academic collaboration

**DOI:** 10.3389/fmed.2026.1870267

**Published:** 2026-07-16

**Authors:** Donatus Rutajama Mutasingwa, Nancy Matillya, Enica Richard, Matilda Alfred Mkonyi, Elichilia R. Shao, Michael Burke, Henry Ziegler, Esther M. Johnston, Klaus B. Von Pressentin, Mugambi Joy, Innocent Besigye, Paschal Ruggajo, Riaz Ratansi, Eric Aghan, Katherine Dominique Rouleau, Davis Rubagumya, Aziza Magram, Florence Salvatory Kalabamu

**Affiliations:** 1 Department of Family and Community Medicine, University of Toronto, Toronto, ON, Canada; 2 Oak Valley Health, Markham, ON, Canada; 3 Department of Family Medicine, Aga Khan University, Dar es Salaam, Tanzania; 4 Department of Otorhinolaryngology, Muhimbili University of Health and Allied Sciences (MUHAS), Dar es Salaam, Tanzania; 5 Department of Obstetrics and Gynecology, Muhimbili University of Health and Allied Sciences (MUHAS), Dar es Salaam, Tanzania; 6 Department of Internal Medicine, KCMC University School of Medicine, Moshi Urban, Tanzania; 7 School of Public Health, KCMC University School of Medicine, Moshi Urban, Tanzania; 8 Health Tanzania Foundation, Dar es Salaam, Tanzania; 9 University of Washington School of Public Health, Seattle, WA, United States; 10 Department of Family Medicine & Community Health University of Minnesota, Minneapolis, MN, United States; 11 The Global Engagement Network for Primary Health Care (GEN-PHC), Minneapolis, MN, United States; 12 Division of Family Medicine, Department of Family, Community and Emergency Care, University of Cape Town, Cape Town, South Africa; 13 Department of Family Medicine, Kabarak University Nakuru, Nakuru, Kenya; 14 Department of Family Medicine, School of Medicine, College of Health Sciences, Makerere University, Kampala, Uganda; 15 Department of Internal Medicine, Aga Khan University, Dar es Salaam, Tanzania; 16 SEED Global Health, Lusaka, Zambia; 17 Healthcare Centre de Reef, The Hgue, Netherlands; 18 Department of Pediatrics and Child Health, Kairuki University, Dar es Salaam, Tanzania

**Keywords:** advocacy, deliberative dialogue, family medicine, partnership, Tanzania

## Abstract

Tanzania is currently advancing universal health coverage (UHC) through the 2026 launch of its Universal Health Insurance scheme, creating a critical policy window to strengthen primary health care (PHC). This paper reflects on the first Family Medicine Symposium in Morogoro (January 2026), a milestone event designed to advocate for the integration of Family Medicine (FM) into the national health system. Utilizing a deliberative dialogue approach, the symposium convened 51 diverse stakeholders—including Tanzanian policymakers and academic partners from eight countries—to examine FM through the “5 Cs” framework: first-contact access, continuity, comprehensiveness, coordination, and person-centered care. The event featured expert presentations, case vignettes, and facilitated discussions structured around the 5 Cs. In addition to the 5 Cs we also discussed training, financing, and workforce transitions. We describe a unique, multinational, multidisciplinary and informal academic partnership that drove this initiative. Rather than following rigid, highly resourced programmatic frameworks, this collaboration was relationship-driven, built on 3 years of trust-building and the contextual expertise of international partners with prior lived experience in Tanzania. While the absence of immediate formal policy commitments remains a limitation, the engagement established a vital transnational platform for collective advocacy. This paper highlights how informal, context-sensitive partnerships can align FM educational initiatives with health system needs to advance PHC transformation.

## 1 Introduction

While academic collaborations to strengthen primary care are increasing (1), few address the critical linkage between professional education and the health systems expected to absorb graduates. This disconnect often results in underutilized professionals unable to practice to their full scope. Strengthening these connections is vital for improving health system performance. The Lancet Commission on Education of Health Professionals for the 21st Century (2) underscores that training primary care professionals is only effective when health systems generate sufficient demand to attract and retain them in rewarding roles.

Previous literature often characterizes collaborations focused on education and capacity building as “high value,” when they possess substantial funding, formal governance, and long-term institutional commitments (3). While these features support scalability and sustainability, such categorizations may overlook flexible, context-responsive partnerships. Funding magnitude and formal structures do not necessarily capture relationship quality, local ownership, or adaptability. Consequently, smaller-scale or informal collaborations risk remaining under-recognized despite their potential for meaningful contribution.

This paper highlights an informal collaboration model, illustrating how impact can emerge through shorter-term, relationship-driven engagements that prioritize trust, mutual learning, and responsiveness to local priorities. Unlike rigid programmatic frameworks, such collaborations evolve organically, allowing partners to align around shared goals and respond to emerging opportunities in real time. We reflect on a recent informal academic collaboration that supported collective advocacy for family medicine in Tanzania during the First Family Medicine Symposium in Morogoro, Tanzania. This engagement brought together local and international stakeholders in a relatively short timeframe, creating space for dialogue, agenda-setting, and the strengthening of professional networks. While modest in scale and duration, the collaboration facilitated alignment around the role of family medicine within the national primary health care agenda and contributing to the discipline’s ongoing recognition and support.

### 1.1 Context

Tanzania has population of approximately 61.7 million with over 60% under age 25 (4, 5). The country is undergoing an epidemiologic transition, where non-communicable diseases (NCDs) now account for roughly 33% of total deaths, reflecting a significant shift from the historical predominance of infectious diseases (6). This dual burden of NCDs and communicable diseases presents important challenges for the health system.

The health system is organized into a pyramidal structure of primary, secondary, and tertiary levels. Primary care is the frontline, with over 11,800 facilities; dispensaries alone comprise 70% of the total network. While the public sector owns most facilities, private and faith-based organizations provide a large share of higher-level district and regional care (7–9). Despite this structure, care coordination remains suboptimal. Weak referral mechanisms lead to service duplication, increased costs, and inappropriate utilization of tertiary centers (9, 10).

The Tanzanian health system has traditionally been characterized as hospital-centric, with relatively underdeveloped community-oriented primary care. However, recent efforts by the Government of Tanzania have focused on strengthening community-based services through the scale-up of community health workers (CHWs). The national Community Health Strategy (2019–2025) outlines the deployment of CHWs to improve preventive, promotive, and basic curative services, including screening, early identification, and follow-up of chronic conditions, as well as maternal and child health services (11). Tanzania has also undertaken significant reforms to improve access to health services. The Universal Health Insurance Act was passed in 2023 (12), marking a major step toward achieving universal health coverage (UHC). The implementation of UHC was officially launched in January 2026. In addition, the government has deployed specialist physicians to district hospitals to bring essential services closer to communities (9).

Workforce development remains a critical challenge. The World Health Organization emphasizes the importance of a “fit-for-purpose” health workforce aligned with population needs (13). In Tanzania this is also clearly articulated in the National Health Policy, which emphasizes flexibility in training of the healthcare workforce according to the “changing needs” of the population (14). Global evidence suggests that family physicians can strengthen health systems by improving the “5 Cs” first contact access, continuity, comprehensiveness, coordination, and person-centered care (15, 16). These attributes are particularly important in settings like Tanzania, where care remains fragmented and often organized around vertical, disease-specific programs.

A review of local, regional, and global policy frameworks underscores the urgency of strengthening primary care in Tanzania. The Health Sector Strategic Plans (HSSP I–V) consistently highlight the need to improve referral systems, enhance workforce capacity, and strengthen primary care delivery (9). Tanzania Development Vision 2025 similarly emphasizes human capital development and access to quality health services as key national priorities (17).

The new Vision 2050, which builds on Vision 2025, and places a stronger emphasis on building a high-quality, resilient, and equitable health system capable of addressing both communicable and non-communicable diseases within a rapidly growing and urbanizing population (18). Vision 2050 underscores the importance of strengthening primary health care, improving health system efficiency, and investing in a skilled health workforce as central pillars for achieving improved population health outcomes and sustainable development. Regionally, African Union Agenda 2063 mirrors these goals, calling for integrated NCD management and PHC infrastructure investment (19).

This paper examines how family physicians can address health system gaps and support UHC goals in Tanzania. It details the Morogoro Family Medicine Symposium, which convened stakeholders to explore policy options and to further define and contextualize the role of family medicine within the Tanzanian health care system. Finally, it reflects on how collaborative, dialogue-driven engagements can support the integration of family medicine into national primary health care strategies.

## 2 Symposium design

### 2.1 Deliberative dialogue

Unlike traditional conferences that rely primarily on expert-led presentations, this symposium adopted a structured approach that combined evidence-informed presentations, facilitated small-group discussions, and plenary synthesis. Specifically, we utilized a deliberative dialogue (DD) approach, a participatory method that integrates research evidence with stakeholder perspectives to support collaborative decision-making. This approach is particularly well suited to complex, policy-oriented health system challenges, where diverse stakeholders—including policymakers, clinicians, researchers, and community representatives—must engage in informed dialogue and reach consensus on feasible implementation strategies.

Deliberative dialogue convenes key stakeholders in a facilitated process to examine available evidence, incorporate experiential knowledge, and collectively identify contextually appropriate solutions (20, 21). Unlike traditional consultation models, DD emphasizes mutual learning, critical reflection, and the co-construction of knowledge, thereby enhancing the relevance, acceptability, and uptake of proposed policy and system interventions.

DD has been widely applied across health systems for multiple purposes, including: supporting knowledge translation and policy advocacy (20, 21); identifying and refining feasible policy options in complex areas such as public health and social policy (22); and co-designing programs and implementation strategies that are responsive to community and system needs (23). Its use is particularly valuable in contexts where bridging the gap between evidence, policy, and practice is essential, and where reforms must be both context-sensitive and stakeholder-informed.

### 2.2 Academic partnership

The symposium was organized by an informal, multidisciplinary multi-institutional collaboration comprising partners from Tanzania, Eastern and Southern Africa as well as North America (Canada and USA) as well as Europe (Netherlands). This group identifies as the Tanzania Family Medicine Development Task Force and has been meeting regularly for the past 3 years to work on developing family medicine curriculum in Tanzania and have shared its work through a publication (24).

The organizing committee was responsible for overall planning and execution of the symposium. Key responsibilities included: developing the symposium agenda; identifying, inviting, and coordinating speakers and participants; and securing financial and logistical support. The committee also prepared all supporting materials, including conference posters, workshop flyers, and participant information pamphlets.

This collaborative structure enabled coordination across institutions and sectors, reflecting the broader goal of fostering shared ownership and collective advocacy for the advancement of family medicine in Tanzania.

### 2.3 Framework for discussion

The symposium deliberations were anchored in the “5 Cs”(see Table 1) of high-quality primary care, a widely recognized framework for evaluating and strengthening health systems (25). This foundational structure was augmented by other important cross-cutting themes including: the training of family physicians, the rationale for investment in family medicine, financing mechanisms, and workforce transitions. The 5C framework was the primary focus while training, financing, and workforce transitions were discussed thematically but not evaluated. Given the importance of multi-level alignment, the symposium also emphasized collaboration with regional and global partners, highlighting the concept of collective advocacy in advancing primary health care and family medicine.

**Table 1:** 5 Cs of primary care (15).

Concept	Definition
First-contact access	Ensuring that the health system provides an accessible and appropriate initial point of care.
Continuity of care	Fostering sustained, longitudinal relationships between patients and providers.
Comprehensiveness	Delivering a broad range of health services within a single care setting.
Coordination	Effectively managing patient care across different levels and providers within the health system-including effective referrals.
Person-centered care	Prioritizing the individual’s needs, preferences, and context rather than focusing solely on disease.

Reflecting the vital intersection of policy design and implementation, each day was formally opened by representatives from the Ministry of Health or the President’s Office – Regional Administration and Local Government (TAMISEMI). The participation of these two government authorities underscored a comprehensive approach to health system, bridging the gap between policy development (Ministry of Health) and service delivery and policy implementation (TAMISEMI).

### 2.4 Ethical statement

Post-event feedback was collected anonymously. Survey completion implied consent, and no identifiable data were recorded.

### 2.5 Symposium implementation, participation, and knowledge translation

Participants represented a broad range of stakeholders across policy, academic, and clinical domains from collaborating institutions and beyond. The event convened a diverse cohort of 51 stakeholders. Of these, 36 engaged in person and 15 participated virtually, representing Tanzania and 10 additional countries, including Kenya, Uganda, South Africa, Malawi, Zambia, Canada, the Netherlands, the United States, Australia and UK.

The participant roster reflected a multi-sectoral approach to health system strengthening. Key national bodies included the Ministry of Health, TAMISEMI, the National Health Insurance Fund (NHIF), the Medical Council of Tanganyika (MCT), and the Medical Association of Tanzania (MAT). These were joined by academic leaders from local institutions—such as KCMC and Kairuki University—and international partners. International partners included experts from Canada (University of Toronto), the Netherlands, the United States (University of Minnesota), Malawi, Uganda (Makerere University), Kenya, and South Africa (the University of Cape Town), UK and Australia.

To facilitate this broad gathering, the symposium was hosted in Morogoro. This location was selected for its strategic position between Dar es Salaam and Dodoma, offering high-speed rail accessibility to ensure maximum attendance from both commercial and political stakeholders.

The proceedings were managed by a dedicated facilitator to maintain structured dialogue and temporal efficiency, while designated recorders systematically documented all discussions. The agenda (detailed in Tables 2, 3) balanced expert presentations with a heavy emphasis on interactive engagement.

**Table 2:** Program schedule: family medicine symposium-day 1.

Topic	Approach/type	Significance	Time (minutes)
Thursday morning session			
Opening Remarks and Introductions	Presentation	Introduction	5
Historical Overview of Family Medicine in Tanzania	Presentation	Background and setting	15
Untapped Opportunities for Family Physicians	Presentation	Regional/Alumnus perspective	5
Primary Health Care in Tanzania: Lessons and Opportunities	Presentation	African regional perspectives	5
Plenary I: Evidence for Family Medicine in Africa	Plenary Session	Evidence-based practice	15
Remarks of Guest of Honor: Family Medicine and UHC	Presentation	Ministry of Health perspective	15
Questions and Group Photos	Interactive	Participant engagement	10
Continuity of Care & First Contact: Current State	Panel Discussion	Panelists as catalysts	45
Continuity of Care & First Contact	Small Group	Deliberative dialogue	30
Continuity of Care & First Contact	Synthesis	Reflections	45
Thursday afternoon session			
Plenary II: Strengthening Health Systems Globally	Presentation	WHO/Global perspective	10
Policy Implementation of Family Medicine in Kenya	Presentation	Neighboring country perspective	10
Questions and Group Photos	Interactive	Participant engagement	10
Comprehensiveness: Scope, Gaps, and Referrals	Panel Discussion	Panelists as catalysts	45
Comprehensiveness	Small Group	Deliberative dialogue	30
Comprehensiveness	Synthesis	Reflections	45

**Table 3:** Program schedule: family medicine symposium-day 2.

Topic	Approach/type	Significance	Time (minutes)
Friday morning session			
Opening Remarks and Introductions	Presentation	Introduction	5
Day 1 Overview	Presentation	Setting the agenda for Day 2	10
Measuring What Matters: Primary Care Assessment in Africa	Plenary session	Provide locally relevant evidence	15
Remarks of Guest of Honor: (Implementation of integration of Family Physicians in Tanzanian Health System)	Presentation	President’s Office, Regional Administration and Local Government Tanzania	10
Questions and Group Photos	Interactive	Participant engagement	10
Care Coordination Referral pathways. Coordination gaps.	Small Group	Deliberative dialogue	30
Care Coordination Referral pathways. Coordination gaps.	Synthesis	Reflections	45
Care Coordination Referral pathways. Coordination gaps.	Synthesis	Reflections	45
Community-Oriented & Person-Centred Care	Small Group	Deliberative dialogue	30
Community-Oriented & Person-Centred Care	Synthesis	Reflections	45
Community-Oriented & Person-Centred Care	Synthesis	Reflections	45
Friday afternoon session			
Training Family Medicine Doctors: Why It Matters – Why Now?	Panelist (African Academic Institutions)	Panelists as Catalysts-modes of training/success stories	20
Overview of National Health Insurance Fund (NHIF)	Presentation	Implementation of UHC	10
Action Planning, Commitments & Next Steps	Group discussions	Participant engagement and commitment to action	35

Post-event evaluation indicated a high degree of perceived impact among participants. For instance, the majority of respondents (*N* = 21) agreed or strongly agreed that the symposium improved their understanding of the “5 Cs” and successfully supported the objectives of Universal Health Coverage (UHC). Detailed evaluation data are provided in Supplementary Table 1.

To ensure the deliberations moved beyond the meeting room, a comprehensive knowledge translation strategy was employed: Immediate Dissemination: The Ministry of Health Media Team conducted daily interviews with participants for timely social media updates.

Policy Influence: A dedicated writing team synthesized the discussions into a summary report, which the TAFP is currently transforming into policy briefs for government distribution.

Academic Continuity: Members of the organizing committee have committed to presenting these findings at upcoming national conferences to maintain momentum for family medicine’s role in the Tanzanian health landscape.

## 3 Discussion

This paper reports on and reflects upon the first Family Medicine Symposium in Tanzania, which aimed to provide an interactive forum between policymakers and champions of family medicine. Through the deliberative dialogues design, the symposium functioned simultaneously as a platform for knowledge translation, awareness-building, and strategic communication. In addition, it served as an important space for relationship-building between family medicine advocates and policymakers. Previous studies have described symposia and conferences as effective “communication channels” for disseminating evidence (26), and have shown that strengthened communication can enhance the uptake of evidence in policymaking processes (25). More broadly, there is growing recognition of the need for sustained collaboration among stakeholders to advance evidence-informed policymaking (27).

One key observation from the symposium was the need for ongoing dialogue among stakeholders, alongside sustained advocacy, to deepen understanding of the role of family medicine in advancing Universal Health Coverage (UHC) in Tanzania. Policymakers emphasized the importance of framing communication in a constructive manner that promotes understanding of the health system rather than criticism of its existing structures. Striking this balance- “making them understand rather than criticizing them”-emerged as both an important principle and a practical communication challenge for advocates of family medicine.

Another emerging theme was the question: “Who is a family doctor?” This reflected broader issues of professional identity and role recognition. Notably, there is currently no widely used equivalent term in the local Swahili language, a gap that was highlighted during small group discussions and has been identified in a recent publication (28). This ambiguity is mirrored among local medical specialists, who have noted that a general lack of awareness makes it difficult to differentiate family physicians from other general practitioners or internists (28). Although approximately 23 family physicians are currently practicing in Tanzania-most of them based in Dar es Salaam-professional identity remains an important but not sufficient factor in strengthening recognition of the discipline. Previous studies emphasize that the establishment of family medicine is influenced by a combination of synergistic factors, including the presence of champions, committed partnerships, and enabling policy windows (1). Following this symposium, members of the newly formed Tanzania Association of Family Physicians (TAFP) recommended *“Daktari Bingwa wa Afya Msingi”* as the Swahili term for “family doctor,” translating directly as “specialist in primary care.”

The symposium coincided with the Government of Tanzania’s official launch of a Universal Health Insurance scheme as part of its broader UHC agenda, representing a timely policy window. However, whether this engagement will translate into concrete policy adoption of family medicine remains uncertain. Evidence from similar academic conferences suggests that direct policy uptake is often limited, as illustrated in experiences from India (29).

The symposium also demonstrated how informal, multi-disciplinary and multi-institutional partnerships can effectively advance a shared agenda while fostering collective learning. The organizing team comprised members from multiple institutions across several countries who had collaborated over the preceding 3 years. This sustained engagement allowed time for trust-building, the development of shared norms, and a deeper appreciation of the local context in Tanzania. These longitudinal relationships enabled more efficient coordination and more open exchange of ideas, while also reducing the transactional dynamics that can characterize short-term collaborations. Trust-based relationships have consistently been identified as a critical determinant of effective global health partnerships, enabling transparency, mutual accountability, and the ability to navigate differences in priorities and perspectives (30).

Furthermore, the intentional recruitment of partners with prior lived experience in Tanzania further strengthened the collaboration. All international members of the working group had previously lived and worked in Tanzania for at least 1 year, contributing not only technical expertise but also contextual knowledge of the health system, policy environment, and professional culture. This prior engagement enhanced credibility with local stakeholders and supported more contextually grounded contributions. It also helped mitigate common challenges in North–South collaborations, including misalignment with local priorities and limited understanding of implementation realities. Collectively, these relational and experiential dimensions contributed to a partnership model that was adaptive, context-sensitive, and locally responsive, reinforcing the potential of informal collaborations to advance family medicine development and primary health care strengthening.

As highlighted in participant feedback, the intentional inclusion of family physicians from neighboring countries was particularly valuable. Policymakers expressed a preference for learning from health systems that are geographically, culturally, and economically comparable, underscoring the relevance of regional experience in shaping policy dialogue. Such approach is not uncommon and was previously used in both Zimbabwe and Botswana during as they were developing their family medicine programs (31).

This reflection is limited by the lack of a rigorous evaluation of symposium uptake specifically among policymakers, as results were aggregated across all participants. Additionally, no formal policy commitments were secured from government officials, likely due to the representation of lower-ranking delegates from the two ministries involved. It should be noted that higher ranking officials were invited but had last minute cancelations due to other more urgent Government priorities. Nevertheless, participation of lower ranking delegates represents an important milestone in increasing awareness and sustaining engagement toward the integration of family medicine within the national health system. Future similar symposiums may engage in securing a signed statement or declaration of support without necessary getting political commitments.

While impactful, this informal partnership faces structural limitations. These include limited long-term sustainability, an overreliance on individual champions, and a lack of institutional memory. The network could address these gaps by formalizing its structure, drawing inspiration from established regional and global networks like AMPATH^1^, PRIMAFAMED^2^, or GEN-PHC^3^.

The core strength of this symposium lies in its reinforcement of the bidirectional link between education and health systems. By engaging both educators and policymakers in mutual learning, the event helps refine family physician training while fostering their formal integration into future practice environments. Furthermore, the agenda prioritized interactive dialogue over static presentations, ensuring ample time for meaningful discussion and shared reflection among all participants.

Furthermore, this analysis underscores the importance of engaging leadership across multiple levels-local, national, and global-as a key enabler of successful education reform and health system strengthening. It also highlights a persistent misalignment between the development of family medicine training programs and broader health system readiness to absorb and utilize family physicians effectively. Addressing this gap will be essential to fully realizing the potential of family medicine as a cornerstone of a strengthened primary health care system in Tanzania.

In conclusion, this analysis contributes to the growing body of literature on ongoing efforts to establish and strengthen family medicine in Tanzania and comparable settings. It highlights key lessons from an emerging model of academic-policy engagement and informal partnership. It is hoped that these experiences may inform and support similar initiatives in other contexts seeking to advance primary health care transformation.

## Data Availability

The original contributions presented in the study are included in the article/Supplementary material, further inquiries can be directed to the corresponding author.

## Footnotes

1
https://www.ampathkenya.org/

2
https://www.primafamed.uraweb.be/

3
http://www.genphc.org/
