# Supplementary material for: Introducing family medicine in Tanzania: strengthening primary health care through the 5 Cs—advocacy through a deliberative dialogue symposium-a case study of multinational academic collaboration
Source: Front Med (Lausanne). 2026 Jul 16;13:1870267. doi: 10.3389/fmed.2026.1870267 (PMC13422557; doi:10.3389/fmed.2026.1870267)
Supplement: Supplementary file 1 [file Table_1.docx]

Supplementary Table

Table : A snapshot of participants’ reflections on the most useful aspects of the symposium included:

| *“Networking, sharing of experiences, and the highly relevant mix of delegates to ensure appropriate stakeholder representation. Also, good involvement of residents and young family doctors. Thanks!”* |
| --- |
| *“To realize the position of family medicine in Tanzania, differences with general practice, and the way forward to improve.”* |
| *“Many lessons learned—I was impressed by the Kenyan example… many points to learn.”* |
| *“The symposium was most useful for strengthening my understanding of comprehensive and coordinated primary care, with clear examples and interactive discussions that can be applied in clinical practice.”* |
| *“Evidence from neighbouring countries on Family Physicians.”* |
| *“To see the engagement with public officials and perspectives from other countries and institutions.”* |
